# Effect of propolis and its active component CAPE on HTLV-1 Tax activities

**DOI:** 10.1186/1742-4690-11-S1-P135

**Published:** 2014-01-07

**Authors:** Mahmoud Huleihel, Jenny Shvarzbeyn

**Affiliations:** 1 Department of Virology and Developmental Genetics, Faculty of Health, Ben-Gurion University of the Negev, Beer-Sheva, Israel

## 

HTLV-1 is the etiological agent of an aggressive malignancy of the CD4^+^ T-cells, called adult T-cell leukemia (ATL) and of certain other severe clinical disorders. The viral Tax protein is a key factor in HTLV-1 pathogenicity. A major part of Tax oncogenic potential is accounted for by its capacity of inducing the transcriptional activity of the NF-kB factors, which regulate the expression of numerous cellular genes. Persistent activity of NF-kB factors has been proved to play a central role in the pathophysiology of ATL and other clinical disorders. PE, a natural product produced by honeybees, has been used for long time in folk medicine. One of its active components, caffeic acid phenethyl ester (CAPE), was found to be a potent inhibitor of NF-kB activation. The main aim of this project was to pursue the possibility of blocking all Tax oncogenic effects in the cytoplasm and the nucleus, by treatment with these products. The cells were transected with a plasmid expressing Tax protein and plasmids containing the examined promoters. Our results showed that both PE and CAPE substantially inhibited the activation of NF-kB-dependent promoter by Tax. However, only PE could efficiently inhibit also the activation of SRF- and CREB- dependent promoters by Tax. Also, both tested materials strongly inhibited Tax binding to IkBα and β and prevented their induced phosphorylation and degradation by Tax. However, they were not able to prevent Tax or of NF-kB transport to the nucleus. In addition, only PE prevented NF-kB nuclear activities.

